# Optimizing Crisp Meat Quality with Modified Starches: From Rheological Properties to Post-Freezing Performance

**DOI:** 10.3390/foods14172947

**Published:** 2025-08-24

**Authors:** Can Ouyang, Zhen Zeng, Zhizhi Qin, Jiaqi Ding, Yuntao Liu

**Affiliations:** 1 College of Culinary and Food Science Engineering, Sichuan Tourism University, Chengdu 610100, China; 2 College of Food Science, Sichuan Agricultural University, Yaan 625014, China; 14399@sicau.edu.cn (Z.Z.); diansuniang@icloud.com (Z.Q.); 6220112010@stu.jiangnan.edu.cn (J.D.)

**Keywords:** starch modification, batter functionality, oil uptake reduction, freeze–thaw stability, pre-fried foods

## Abstract

Crisp meat, a traditional Chinese food, is widely consumed due to its convenience and long frozen shelf life. However, conventional preparation methods lead to excessive oil absorption during frying and ice crystal formation during freezing, causing coating softening and reduced crispiness after reheating. This study aimed to enhance the quality of crisp meat before and after freezing by incorporating modified starches into the batter. Four types—oxidized starch, hydroxypropyl distarch phosphate, starch acetate, and acetylated distarch phosphate—were tested at replacement levels of 10–40% for natural potato starch (NS). Results showed that all modified starches improved batter rheology by 20%, increased viscosity and stability during frying, and delayed retrogradation during freezing compared to NS. Among them, 20% acetylated starch has a better effect on improving the quality of frozen small crisp meat for enhancing water-holding capacity, increasing immobilized water content, reducing oil uptake by 12–18%, and improving product texture. Specifically, they helped maintain a crispier coating after reheating, addressing a key drawback of traditional crisp meat. In conclusion, modified starches significantly improved frying performance and minimized quality loss during freezing compared to NS. This study provides practical insights for the food industry in optimizing batter formulations for better-quality crisp meat products.

## 1. Introduction

**Figure 1:**
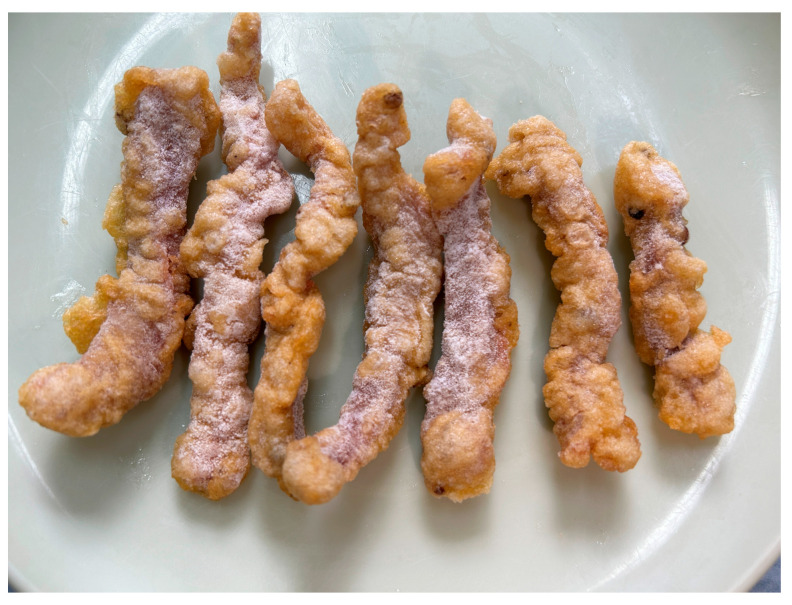
Frozen crispy meat.

Premade cuisine refers to edible produce that has been adequately pre-treated before purchasing and can be consumed directly or after simple heat treatment. In recent years, premade cuisine has undergone rapid development in China. Many traditional delicacies are favored by consumers due to their long shelf life, quick cooking, and convenient consumption when sold as frozen food (e.g., frozen crispy meat). Pre-fried battered foods are pre-processed by battering and partial frying, then stored, circulated, and sold under frozen conditions, and can be consumed after twice-frying. Furthermore, pre-fried foods appeal to the modern population of all ages worldwide, particularly in developed countries, due to their delicious taste, crispy texture, attractive color, convenience, and rapidity; hence, an essential field for research [1]. Frozen crispy meat, as shown in Figure 1, is an example of prepared food that has become increasingly popular among consumers in today’s fast-paced life and the advancement of storage equipment. Frozen crispy meat has surged in popularity due to growing demand for convenient, globally distributed prepared foods, particularly following pandemic-driven shifts in consumption patterns [2,3]. However, its quality deteriorates during freezing through physical processes like ice crystal-induced cellular damage, protein denaturation, reducing water-holding capacity, and starch retrogradation, causing texture hardening [4,5,6]. Deep-frying further introduces health concerns, as high oil absorption contributes to obesity risks, while high-temperature cooking generates carcinogenic acrylamide and pro-inflammatory polycyclic aromatic hydrocarbons [7,8,9]. Moreover, color and flavor decline after twice-frying have become the two major problems in traditional, frozen, pre-fried battered foods, contrary to current industry trends prioritizing reduced oil absorption, extended frozen stability, and sensory quality [10]. Thus, it is critical to prevent quality degradation in prepared foods during frozen storage and frying. Numerous studies have reported that the batter quality of pre-fried foods can be enhanced by adding modifiers [11,12,13,14].

Industry innovations like modified starch batters (to inhibit ice crystals) and air-frying technologies are being developed to address these challenges while meeting consumer demand for convenient, better-quality products. Modifiers, as chemically modified starches, are additives designed to improve batter functionality during processing and storage. The batter coating characteristics of pre-fried crisp meat largely determine the changes in oil and moisture content, flavor, and color of fried foods before and after freezing. Therefore, adding appropriate modifiers to the batters effectively prevents the decline of crisp meat quality [15].

Currently, starch is gaining attention due to its various food applications. Starch is widely used for food processing because of its significant influence on structural properties [10]. Despite that, starch possesses undesirable characteristics that hinder the broader application of this ingredient, such as low rheological stability, poor resistance to machinery and heat, and long-term retrogradation. Consequently, modified starch is gradually replacing native starch in the food industry. Starch modification refers to the alterations of the physical and chemical properties of native starch to improve the functional properties for food and industrial applications [16]. The modifications could be physical, chemical, enzymatic, or a combination of these methods. The chemical is one of the most valuable modifications due to introducing new functional groups through acetylation, cross-linking, oxidation, and substitution to obtain a single-modified starch or double-modified starch with improved viscosity, high temperature, and retrogradation resistance. For example, the oxidized cassava starch could enhance the fried expansion rate of starch [17]. Furthermore, the incorporation of acetylated rice starch at a particular percentage boosted the overall acceptability of fried dumpling skins; therefore, chemically modified starch has become increasingly relevant in frozen pre-fried foods to enhance the frying characteristic and freezing quality of crisp meat and other products.

Previous studies mainly aimed to improve the batter processing properties, such as crispness, color, and oil content of freshly fried foods by modifying starches, including sago sodium triphosphated starch, succinylated and oxidized barley starch, acetylated potato starch, or enhance the stability of frozen foods [18,19,20]. While these studies have examined frying or freezing individually, systematic evaluations of both processes remain limited. Therefore, four types of modified starches were selected in this study to replace the native potato starch in gradient concentrations to improve batter properties. Furthermore, the performance of different batters was compared, and the relationship between the changes in batter properties and crisp meat qualities before and after freezing was analyzed. The mechanism of modified starches that could enhance the qualities of fried crisp meat was further elucidated, which provided some theoretical reference to increase the quality of frozen pre-fried battered food.

## 2. Materials and Methods

### 2.1. Materials

Oxidized starch (OST), hydroxypropyl distarch phosphate (HPDSP), starch acetate (CAS), and acetylated distarch phosphate (ADSP) were provided by Starpro Starch Co., Ltd. (Hangzhou, China). Meanwhile, the native potato starch (NS) was supplied by Anjoy Foods Co., Ltd. (Ziyang, China). The Bakerdream wheat flour (medium gluten) was purchased from Angel Yeast Co., Ltd. (Yichang, China), and petroleum ether (boiling range 30~60 °C) was purchased from Jinshan Chemical Test Co., Ltd. (Chengdu, China). The sunflower oil, eggs, pork (chilled fresh pork, exhibited a bright red color, possessed the characteristic fresh pork aroma with no detectable off-odor, displayed a moist surface, showed good elasticity, and had a pH value of 5.6), salt, monosodium glutamate, chili powder, complex phosphate and pepper powder were purchased from a local supermarket. All chemicals and reagents used in this study were of analytical grade unless stated otherwise.

### 2.2. Batter Preparation

The powder base (flour/starch = 3:2) was mixed with the egg pulp at a ratio of 1:1.5 and stirred well in the same direction. The modified flour, consisting of OST, HPDSP, CAS, and ADSP, was included at different concentrations (0%, 10%, 20%, 30%, and 40%) as partial substitutes for NS. The series of batter concentrations was as follows: NS, OST-10%~OST-40%, HPDSP-10%~HPDSP-40%, CAS-10%~CAS-40% and ADSP-10%~ADSP-40%.

### 2.3. Meat Preparation

The method for preparing crisp meat in this study was developed based on the Chinese industry standard T/AHFS 003-2022 (Prepared Meat Dishes—Crisp Meat) [21], with specific modifications to align with our experimental design. The pork tenderloin was cut into uniform strips and marinated at 4 °C for 24 h. The meat marinate was prepared according to the following formulation: 2% salt, 0.4% monosodium glutamate, 0.4% pepper powder, 0.5% chili powder, 0.8% compound phosphate, and 15% water. The proportion of ingredients was calculated according to the total weight of the meat.

### 2.4. Rheological Measurements

The rheological behaviors of the modified batter were determined using a rheometer (DHR-1, TA Instruments, New Castle, DE, USA) with a stainless-steel parallel plate (40 mm in diameter) and a gap size of 1000 μm for all experiments. All the batters were prepared as stated in Section 2.2 and allowed to rest for 1 h prior to the experiment.

#### 2.4.1. Dynamic Scanning Tests

The dynamic rheological tests were initiated by ramping the temperature from 25 °C to 80 °C at a 2 °C/min heating rate. The changes in storage modulus (G′), loss modulus (G″), and loss tangent (tan δ = G″/G′) during heating were recorded.

#### 2.4.2. Steady-State Flow Tests

The steady-state flow tests were performed at a temperature of 25 °C with continuous shearing of 0.1–100 s^−1^. The starch paste viscosity often displays the non-Newtonian feature: the shear stress does not increase linearly with the increase in shear rate; thus, the experimental curves were in accordance with the power law model [22]:

$$\tau \;=\;K\overset{•}{\gamma }n$$

where τ is the shear stress (Pa), K is the consistency coefficient (Pa·sn), $\overset{•}{\gamma }$ is the shear rate (s^−1^), and n is the flow behavior index (dimensionless) [23]. The coefficient R^2^ is applied to evaluate the model fitting.

### 2.5. Batter Pick Up

The batter hanging rate represented the total amount of batter attached to the meat, which was expressed as the ratio of the weight of the attached batter to the total weight of the meat after coating. The meat core was immersed in the batter for 15 s, then removed and left to drip for 10 s. The experiment was conducted in triplicate. The batter’s hanging rate was calculated as follows:

$$\mathrm{Batter}\;\mathrm{pick}\;\mathrm{up}\;(\%)\;=\;(\frac{B}{B+S})\times 100$$

where B is the weight of the attached batter, and S is the weight of the meat core.

### 2.6. Gelatinization and Retrogradation Properties

The thermodynamic properties of the batters were determined using the differential scanning calorimeter (Q200, TA Instruments, USA). First, the samples (10 mg) were transferred into an aluminum pan (Shanghai Jingyi Chemical Materials Co., Ltd., Shanghai, China) and sealed immediately. An empty aluminum pan was used as a reference. Subsequently, the gelatinization characteristic procedure was initiated when the temperature rose from 20 °C to 170 °C at a heating rate of 10 °C/ min and performed thrice for each sample to evaluate its thermodynamic properties The indicators included the onset (To), peak (Tp) and conclusion (Tc) temperatures and the enthalpy of gelatinization (∆Hg). After cooling, the samples were stored at 4 °C for 10 d before the second measurement. Finally, the retrogradation enthalpy (∆Hr) was determined by increasing the temperature from 20 °C to 230 °C at a rate of 10 °C/min.

### 2.7. Frying Conditions

The frying of samples was performed in an automatic temperature-controlled fryer (WEIGHMAX Co., Los Angeles, CA, USA) with 500 mL of sunflower oil. The samples were pre-fried in hot oil at 160 °C for 25 s and divided into two batches. One batch was refried at 180 °C for 3 min to prepare freshly fried crisp meat, while the other batch was cooled rapidly with liquid nitrogen and frozen at −18 °C for 10 d before being thawed for 1 h and refried at 180 °C for 3 min. At the end of the frying period, the samples were removed from the fryer basket and blotted with tissue paper. The samples were allowed to cool at room temperature before oil and water content analyses [24].

### 2.8. Determination of Moisture Content

Moisture in the meat core of fried foods is a key factor in maintaining the overall juiciness and tenderness; hence, the moisture content analysis in this study. First, the meat core was cut into small granules and weighed (1.5 g). Subsequently, the meat granules were dried in a moisture meter (XY-105W, Qingdao Toky Instruments Co., Ltd., Qingdao, China) at 105 °C until a constant weight was achieved. Finally, the moisture content was expressed as the percentage of sample mass after drying to the sample mass before drying.

### 2.9. Determination of Oil Content

Studies have reported that most of the oil in fried foods is absorbed into the product surface or drawn into the porous shell microstructure during cooling [25]. Therefore, the shell was selected for oil content measurement. The oil content in the shell of fried crisp meat was determined using the crude fat tester (SZF-06A, Shanghai Xinjia Electronic Co., Ltd., Shanghai, China). First, the shell was cut into rice grains, weighed (2.3 g) (M) in a fat extraction bottle, and Soxhlet extracted using petroleum ether for 5 h at 60 °C. After extraction, the remaining solvent was evaporated, and the extraction bottle was oven-dried at 105 °C for 1 h. Subsequently, the sample was cooled in a desiccator for 30 min before weighing. This step was repeated until a constant sample weight was achieved. The oil content in the shell was expressed percentage and calculated using the following equation:

$$\mathrm{Oil}\;\mathrm{content}\;(\%)\;=\;\frac{M_{2}-M_{1}}{M}\times 100$$

where M represents the weight of the weighed shell (g), M_1_ represents the weight of the fat extraction bottle (g), and M_2_ represents the total weight of the extraction bottle and oil after constant weight (g) was achieved.

### 2.10. Texture Profile Analysis (TPA)

The meat crispness was estimated at room temperature using a texture analyzer (TA-XTPlus, Stable Micro System Ltd., Leicestershire, UK) with a probe (P/36r). The texture parameters were set as the pre-test, test, and post-test speed of 2 mm/s, 1 mm/s, and 1 mm/s, respectively, with a compression degree of 50%, two compression cycles, 5 g trigger force of 5 g, and 5 s interval.

### 2.11. Color Analysis

The color of the crisp meat shell was determined using a portable multifunctional chromameter (NR60CP, Shenzhen Threenh Technology Co., Ltd., Shenzhen, China) calibrated with a referenced white plate. The results were recorded as L*, a*, and b* based on the CIE (Coherent Infrared Energy) Lab system, where L* reflects lightness from black (0) to white (100), a* and b* describe green (−) to red (+) and blue (−) to yellow (+), respectively [26].

### 2.12. LF-NMR Analysis

#### 2.12.1. Determination of T2 Relaxation Time

LF-NMR experiments were carried out using an NMR analysis system (NMI20-060H-I, Suzhou Niumag Analytical Instrument Co., Suzhou, China) with a magnetic field strength of 0.5 T, a magnet temperature of 32 ± 0.01 °C, a coil diameter of 60 mm, and a proton resonance frequency of 21 MHz. First, the T2 transversal relaxation curves were measured using the Carr–Purcell–Meiboom–Gill (CPMG) sequence. The parameters used in the CPMG sequence were set as follows: sampling repetition time, TW = 1500 ms, number of repeated samples, NS = 32, echo time, TE = 0.2 ms, number of echoes, NECH = 2000, and preamplifier gain, PRG = 1. Then, the sample wrapped with the plastic film was placed in glass tubes with a diameter of 40 mm, before being transferred into the NMR instrument for analysis to obtain the exponential attenuation spectrum. Finally, the inversion spectrum, T2, was recorded using the inversion software MultiExp Inv Analysis (Suzhou Niumag Analytical Instrument Co., Suzhou, China) [27].

#### 2.12.2. Determination of Magnetic Resonance Imaging (MRI)

Spin-echo (SE) imaging sequence was used to obtain the T2-weighted images of crisp meat. The field of view (FOV) for all images was 100 × 100 mm, repetition time, TR = 1700 ms, echo time, TE = 19 ms, slice count = 5, slice width = 4 mm, slice gap = 2 mm, average = 4.

### 2.13. Statistical Analysis

All data were expressed as mean and standard deviation. Unless specified, each experiment was replicated independently in triplicate. SPSS version 26.0 was used to perform the two-way analysis of variance (ANOVA), Tukey’s HSD post hoc tests to make all pairwise comparisons between starch types at each concentration level, and *p* < 0.05 indicated a statistically significant difference.

## 3. Results and Discussion

### 3.1. Rheological Characterization Analysis

#### 3.1.1. Dynamic Temperature Scanning Analysis

**Figure 2:**
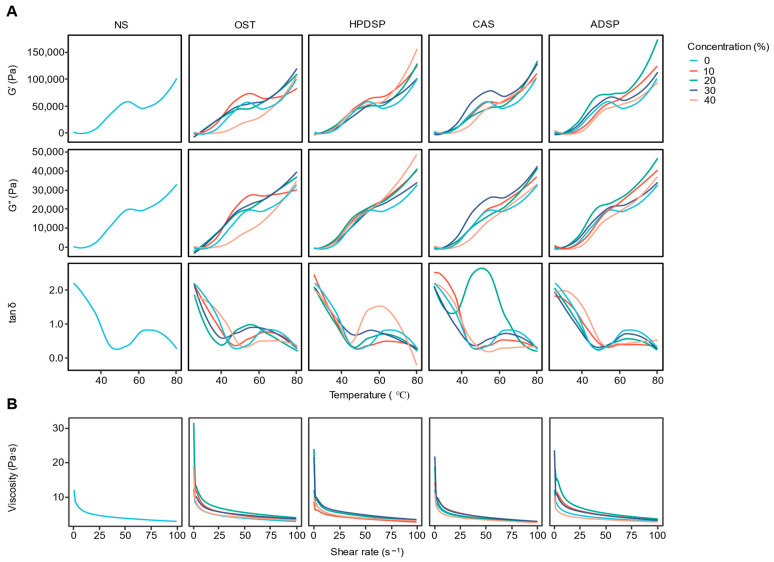
Rheological characteristics of batters with different types and concentrations of modified starches. (**A**): Storage modulus (G′), loss modulus (G″) and loss tangent (tan δ = G″/ G′) of dynamic scanning tests; (**B**): viscosity of steady-state flow tests.

The changes in storage modulus (G′) and loss modulus (G″) of batters prepared using different modified starches during heating are illustrated in Figure 2A. The G′ represents the elastic behavior by measuring the stored energy, while G″ indicates the viscous behavior by measuring the dissipated energy [28]. If the G′ is smaller than G″ (tan δ > 1), the sample behaves more like a liquid. As observed in Figure 2A, during the initial heating stage, the storage modulus (G′) and loss modulus (G″) of all batters generally continued to increase, with G″ exceeding G′ (tan δ > 1), indicative of a liquid-like system. Notably, among these samples, OST 40 and CAS 40 exhibited less pronounced growth magnitudes in G′ and G″ during this initial phase compared to other groups; nevertheless, they still maintained an overall upward trend. As the temperature rises, the G′ became greater than G″ (tan δ < 1), and the system began exhibiting solid gel behavior.

During the long heating process, the starch particles were broken and disintegrated, microcrystals melted, and interchain interactions were weakened, thus decreasing G′ and G″ with the rising temperature [29]. The G′ and G″ increased for all modified starches, whereas the G′ for all treatment samples was significantly higher than NS during heating. The amount and type of modified starch significantly impacted the viscoelasticity of the batter, primarily due to differences in their functional group modifications and molecular structures.

At the initial heating stage, NS exhibited lower G′ and G″ with G″ > G′ (tan δ > 1), indicating dominant viscous behavior. In contrast, OST showed enhanced G″ growth at low temperatures, strengthening its liquid-like characteristics, while HPDSP and ADSP displayed relatively moderate G″ increments and closer G′/G″ balances, with milder liquid-like behavior. CAS stood out with the lowest initial G′ and G″, reflecting weakened molecular interactions likely from acetyl groups reducing hydrophilicity.

During gelation, all modified starches transitioned to solid-like behavior (G′ > G″) at lower temperatures than NS, with OST, HPDSP, and ADSP forming stronger gels (higher G′ plateaus) due to enhanced network formation. HPDSP and ADSP, containing both hydrophilic (hydroxypropyl, phosphate) and ionic (phosphate) groups, promoted efficient molecular crosslinking via hydrogen bonding and electrostatic interactions, yielding the strongest gels [30,31]. OST’s carbonyl groups increased chain flexibility and fragmentation, accelerating network assembly [32]. CAS, however, suffered from acetyl groups disrupting intermolecular hydrogen bonds, resulting in the weakest gel strength despite earlier gelation initiation [33].

These differences stemmed from how functional groups modified starch structure: polar groups (phosphate, carbonyl) enhanced hydrophilicity and crosslinking, flexible chains (hydroxypropyl) regulated chain mobility, and hydrophobic groups (acetyl) reduced molecular cohesion [34]. Such structural variations directly influenced gelation kinetics and final gel strength, with HPDSP and ADSP excelling in strong gel formation, OST balancing viscous–elastic properties, and CAS showing limited gelation capacity, collectively demonstrating how targeted modifications tailor starch behavior for specific applications.

#### 3.1.2. Steady-Shear Flow Behavior Analysis

Figure 2B demonstrates the flow behavior of batters prepared with different types and concentrations of modified starch under steady shear conditions at 25 °C. The apparent viscosity decreased with increasing shear rate, indicating the shear-thinning behavior for all batters. Similarly, previous studies have reported the non-Newtonian flow behavior of starch samples [35,36,37,38,39]. At a high shear rate, the difference in apparent viscosity between samples was smaller than at a low shear rate, suggesting the structural decomposition and rearrangement caused by the shear rate [40]. Furthermore, the apparent viscosity of the treatment groups was higher than the NS group. This behavior could be due to the introduction of hydroxyl, carbonyl, and carboxyl groups in appropriate amounts through the starch modification, thus promoting the expansion of the granules. At low shear rates, NS and CAS showed relatively higher initial viscosities, whereas OST and HPDSP displayed moderate viscosity build-up, likely due to stronger intermolecular interactions. With increasing shear rate, all samples demonstrated shear-thinning behavior, but the degree varied significantly: HPDSP and ADSP exhibited the most pronounced shear thinning, with viscosity dropping sharply at high shear rates, attributed to their flexible hydroxypropyl chains and ionic phosphate groups that reduced molecular entanglements. In contrast, CAS showed milder shear thinning, possibly due to acetyl groups disrupting intermolecular cohesion. OST displayed intermediate shear-thinning behavior, balancing chain fragmentation (from oxidation) and residual intermolecular forces. Notably, HPDSP and ADSP maintained higher viscosities across shear rates compared to NS, reflecting enhanced molecular alignment resistance, while CAS and OST showed reduced viscosity under high shear, indicative of weaker network structures. These differences highlight how functional groups (hydrophilic/hydrophobic, ionic/non-ionic) and molecular flexibility govern the rheological response of starch-based systems under shear.

The critical temperatures for starch gelatinization are influenced by the type of starch, its modification, and concentration. NS exhibits a prominent loss tangent (tan δ) peak at approximately 55 °C, indicating its typical gelatinization threshold. In contrast, waxy starch (OST), which has a lower gelatinization temperature, shows an earlier peak at around 45 °C. Modified starches—HPDSP, CAS, and ADSP—display elevated gelatinization ranges (55–65 °C) due to structural stabilization from phosphorylation/esterification, which delays molecular network formation during heating. Concentration effects are secondary: although higher starch levels (30–40%) enhance viscoelastic moduli, they only minimally shift gelatinization temperatures. Low (0–10%) and high concentrations primarily alter elastic modulus magnitudes rather than transition thresholds [41].

The stress curves of the samples were fitted using the power law model to obtain the model parameters listed in Table 1. All R^2^ values ranged between 0.9860 and 0.9996 (R^2^ > 0.98), confirming that the shear rate and shear stress data conformed well to the power law model. Two-way ANOVA revealed significant effects of both starch type (F = 15.32, *p* < 0.001) and concentration (F = 28.76, *p* < 0.001) on consistency coefficient (K values), with a significant interaction effect (F = 4.12, *p* = 0.002) indicating that the impact of starch type depended on concentration level. In addition, the K values correlated with the consistency of the system; a larger K value indicates a superior thickening effect [42]. The batters with 20% or 30% modified starches showed higher K values (*p* < 0.05), while the increase in K values was not significant or decreased when the amount of modified starches was 40%. Therefore, the modified starches may possess stronger hydration capacity and a thickening effect at appropriate concentrations, indicating that adding modified starches increases the system consistency. Meanwhile, the K value trends were also consistent with the viscosity changes.

The Power law model is widely used to describe the flow characteristics of pseudoplastic (non-Newtonian) liquid. The flow behavior index, n, is dimensionless, which reflects the closeness to Newtonian flow, where n = 1 corresponds to Newtonian fluid, and the lower n value reflects the higher plasticity of the fluid [43]. The flow behavior index (n values) varied significantly by starch type (F = 8.45, *p* < 0.001) and concentration (F = 12.33, *p* < 0.001), though without interaction effects (*p* = 0.287). HPDSP consistently exhibited higher n values across concentrations, indicating different flow characteristics compared to other starch types. The study findings demonstrated that all flow behavior indices (0.638~0.781) were less than 1.0, suggesting that all starch batters exhibited pseudoplastic behavior. Moreover, the value of n decreased (*p* < 0.05) when the concentrations of modified starch were 20% and 30% compared to NS, indicating that the samples prepared with a suitable concentration of modified starches were more pseudoplastic. Likewise, an earlier study reported that the increase in gum Arabic concentration decreased the n value of cationic tapioca starch [44]. In summary, the four modified starches in this study can impart good rheological properties to batters, enhance the viscosity and consistency of the system, facilitate a more uniform and smooth coating process, and produce greater G′ and G″ at higher temperatures, which could predict the improvement of shell stability in the subsequent frying process.

The significant interaction between starch type and concentration (F = 4.12, *p* = 0.002) can be attributed to molecular reorganization dynamics. Cross-linked starches (e.g., ADSP, HPDSP) form stable three-dimensional networks at moderate concentrations (20–30%) through enhanced hydrogen bonding between phosphate/acetyl groups and water molecules. This optimizes chain entanglement while preventing steric overcrowding (at >30%), explaining the peak consistency coefficients (K) and shear-thinning behavior (n < 1) observed in Table 1 and Figure 2B. Such networks directly contribute to batter stability during frying and freezing by resisting oil penetration and ice crystal damage.

Modified starches, through the modification of their functional groups, have significantly improved paste viscoelasticity, shear properties, and the ratio of high-temperature G′/G″. HDPSP and ADSP excelled in gel strength, while OST balanced viscoelasticity, and CAS showed limited gelation. Optimal concentrations (20 to 30%) of these modified starches effectively enhanced system consistency, making them suitable for applications that require improved shell stability.

### 3.2. Batter Pick-Up Analysis

**Figure 3:**
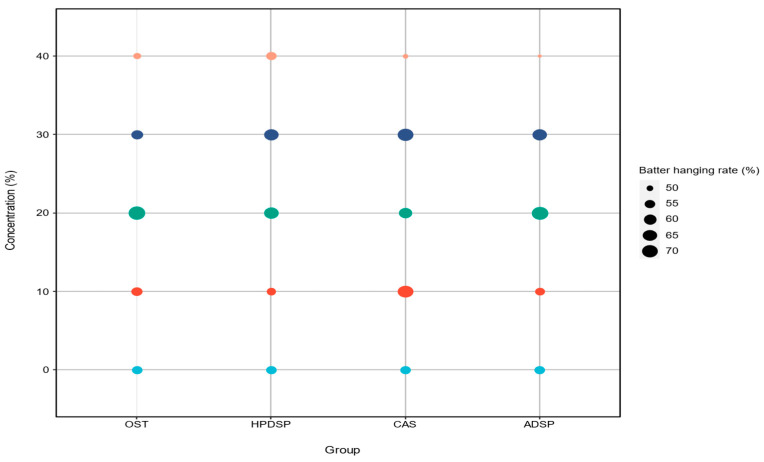
Batter hanging rate of batters formulated with NS and four different types and concentrations of modified starches.

Figure 3 summarizes the effect of modified starch types and concentrations on the batter-hanging rate of crisp meat. The amount of batter attached to the foods before frying is influenced by the viscosity of the batter, a key coating characteristic, and may affect the food’s appearance and texture [45]. When the modified starches were 20% and 30%, the batter hanging rate was significantly enhanced (*p* < 0.05), which showed a good sizing effect and aligned with the trend of rheological properties. In contrast, the batter viscosity decreased when the modified starch concentration was 40%, weakening the coating effect. Therefore, it could be concluded that the changes in batter hanging rate were related to the corresponding viscosity changes.

### 3.3. Gelatinization and Retrogradation Properties Analysis

**Figure 4:**
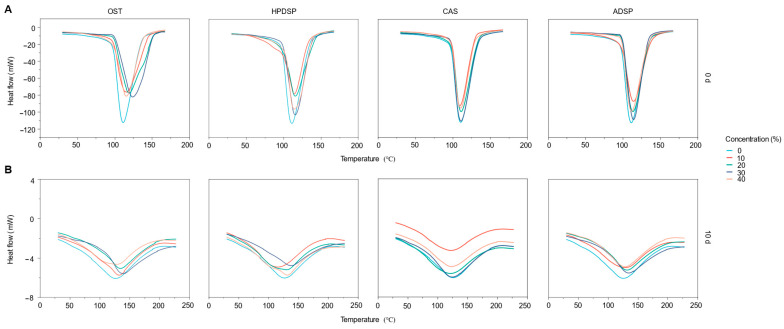
The DSC curves of different batters. (**A**): Gelatinization properties; (**B**): retrogradation properties.

Figure 4 illustrates the DSC curves of batters (NS, OST, HPDSP, CAS, and ADSP) before and after retrogradation. The To, Tp, and Tc of OST, HPDSP, and ADSP exhibited a slight increase compared to NS, while the To, Tp, and Tc of CAS slightly decreased. Based on the findings, large groups such as acetyl and hydroxypropyl may have been introduced in the modified starches, breaking the intramolecular and intermolecular hydrogen bonds and weakening the structure. Conversely, the system was more prone to hydration and expansion due to the strong hydrophilicity of the groups, which promoted the decline of To, Tp, and Tc. Nevertheless, the phosphate ester cross-linking treatment strengthened the structural organization in the starch molecules, limited the expansion of starch particles, and delayed gelatinization, thus making the starch more heat resistant. Therefore, the gelatinization temperature of double-modified starches such as HPDSP and ADSP should consider the combined effect of different treatments.

Oxidation treatment led to the depolymerization of the amorphous region, which alleviated the instability of the crystalline region, leading to an increase in the gelatinization temperature and starch stability. However, the changes in To, Tp, and Tc of different modified starch batters were insignificant relative to the high frying temperature, resulting in contradicting trends in pasting temperature, which was not impactful on the thermodynamic properties. In addition, different groups introduced in the modification process led to the early destruction of the double helix structure in the amorphous region and the hydrolysis of some crystalline regions [46]. Resultantly, lower dissociated and melted double helices were present during the gelatinization process, causing a decrease in ∆Hg compared with NS.

The high-energy and dysregulated starch molecules gradually changed into the low-energy, organized structure during the freezing process. The aggregation and rearrangement between starch molecules via hydrogen bonding to form crystalline polymers led to the decreased ∆Hr of the frozen samples. In addition, modified starches with appropriate concentrations could effectively inhibit starch retrogradation compared to NS, where HPDSP at 30% had the most significant effect. The introduction of hydroxypropyl, acetyl, carboxyl, or carbonyl groups could hinder the reassociation of chains, leading to the reduced tendency of retrogradation and delaying the quality deterioration of pre-fried crisp meat during freezing. This finding was consistent with the study of Sangseethong [47]. Moreover, this result indicated that partial replacement of NS by OST, HPDSP, CAS, and ADSP could alleviate the long-term retrogradation of nature starch gels and potentially extend the shelf life of frozen foods. The introduced hydroxypropyl, acetyl, carboxyl, or carbonyl groups could hinder the reassociation of chains, leading to the reduced tendency of retrogradation. This retardation effect originates from the steric hindrance of functional groups, which competitively bind to water molecules via hydrogen bonding (Figure 4), thereby preventing amylose realignment. Overall, modified starches, particularly those with 30% HPDSP, effectively inhibit retrogradation by impeding chain reassociation, providing theoretical support for optimizing starch formulations in frozen foods and extending shelf life by delaying quality deterioration.

### 3.4. Moisture Content of Crisp Meat

Table 2 demonstrates the moisture content variation in crisp meat at different freezing time points. The moisture content of the samples with 20% modified starch increased compared to NS, but the opposite trend was observed when the modified starch was included at higher concentrations. Furthermore, the water dissipation in the meat core is alleviated to a certain extent due to the introduction of hydrophilic groups such as hydroxypropyl, acetyl, carboxyl, and carbonyl, thus improving the water retention performance [48]. Meanwhile, the rate of reduction in the moisture content of frozen crisp meat was faster than unfrozen samples due to the higher heat transfer between frozen cells during frying caused by the formation of ice crystals from the freezing treatment [49]. Despite that, the moisture content of OST, HPDSP, CAS, and ADSP was still higher than NS, thus indicating the modified starches could mitigate moisture loss during freezing. Additionally, there are strong interaction effects between starch type and concentration (*p* = 0.008 for 0 d, *p* = 0.001 for 10 d). ADSP showed the highest moisture retention at 40% concentration before freezing (61.62 ± 0.43%), and HPDSP demonstrated superior moisture retention after freezing at 30% concentration (58.54 ± 0.45%). In conclusion, all modified starches in this study exerted similar effects and improved the water retention of fried crisp meat when included at 20%. In particular, ADSP at 20–40% concentration demonstrated optimal moisture retention properties, particularly maintaining higher water content after freezing.

### 3.5. Oil Content of Fried Crisp Meat

The hierarchical structure of starch granules changed dramatically during frying from a semicrystalline structure to a more amorphous state, which indicated the disruption of granular morphology, the disintegration of double helices, and the degradation of starch molecules. This transition from ordered to disordered eases the oil molecules to adsorb onto the surface and diffuse through starch granules, thereby allowing the granules to absorb a substantial amount of oil during frying [50]. Adding modified starches decreased the oil content of crisp meat, and the highest reduction rate with the best effect of oxidized starch was recorded at the 20% level of inclusion (Table 3). The esterification, cross-linking, and oxidation reactions in the starch granules contribute to higher resistance to deformation during heating [25] and good film-forming property that forms a dense film that blocks oil penetration during frying [51,52]. In addition, OST at 20% concentration showed the lowest oil absorption (11.86 ± 0.71% before freezing). There are significant differences between starch types that emerged primarily at intermediate concentrations (10–30%). Freezing generally increased oil content for all starch types (*p* < 0.001)

When the modified starch concentrations were increased to 30% and 40%, the oil content of crisp meat increased. The modified starches might have introduced many negative charges that produced strong electrostatic repulsion, easing the destruction of starch granule structure and increasing the oil content. Nevertheless, the balancing trend between oil and moisture content changes is crucial in ensuring good sensory quality, as stated by Zhang [53]. This trend is associated with the moisture substitution mechanism of oil absorption. Food ingredients contain high moisture at the beginning of frying; when the vapor pressure inside the foods exceeds the external environment, the violent moisture escape will prevent the oil from migrating into the samples. As the frying time increased, void formation increased with moisture evaporation, gradually weakening moisture escape. Consequently, more oil penetrates the sample interior through the gaps, thus indicating the negative linear correlation between oil content and moisture content [54]. Therefore, it is essential to understand how to maintain an excellent water–oil balance of frozen prepared foods such as crisp meat.

The oil content of samples frozen for 10 d was generally higher than unfrozen samples, consistent with the changes in moisture content. Nonetheless, the oil content of samples with modified starches was lower than NS. The pre-fried crisp meat produced large ice crystals during freezing, and the internal crystals sublimated directly at high temperatures during frying. As a result, cell destruction, structural damage, and more gap formation occurred, providing sufficient space for oil penetration [55]. The oil content of crisp meat was significantly affected by both modified starch concentration and freezing time. A deeper understanding of the underlying mechanisms—particularly how different freezing conditions influence oil absorption—could enable more effective strategies for reducing oil content in crisp meat products. For oil reduction, oxidized starch (OST) at 20% concentration showed the most effective performance with the lowest oil absorption (11.86 ± 0.71%) in fresh samples. In conclusion, ADSP exhibited the most stable behavior across freezing conditions, making it particularly suitable for frozen storage applications where consistent quality is required. All modified starches, particularly 20% OST with the lowest oil absorption, reduce oil uptake via structural barrier effects, maintaining stability under freezing. Findings highlight strategies to optimize water–oil balance and enhance quality in frozen pre-fried crispy meat.

### 3.6. Crispness of Crisp Meat

**Figure 5:**
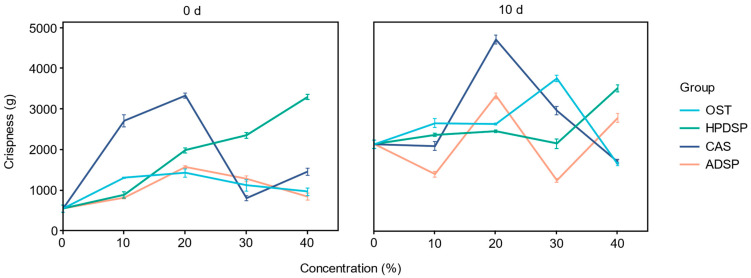
Effects of different types and concentrations of modified starches on the crispness of crisp meat.

Crispness is an important indicator of fried food quality and largely determines consumer acceptability. Figure 5 illustrates the trends of crispness before and after the freezing of fried crisp meat with different types and concentrations of modified starch. The suitable film-forming property of modified starches promoted the film development between the inner core and the outer shell, effectively preventing the diffusion of moisture and oil molecules from the meat to the outer batter. Therefore, adding modified starches improved the crispness of fried crisp meat, hence the crispier texture. After freezing at −18 °C for 10 d, the crispness was further improved because of ice crystal production during freezing, leading to massive water loss during frying. Likewise, when the concentration of modified starch was too high, the crispness of crisp meat decreased, particularly for frozen fried samples. High levels of amylose were modified, resulting in a possible decrease in crispness, as supported by Han [56].

### 3.7. The CIE Color Attributes and Total Color Difference (TCD)

Color is a key quality attribute of fried battered food. The ideal color of fried food is light golden brown, and the increase of parameter a* and the decrease of parameter L* contribute to an intense golden brown color [57,58]. Based on Table 4, more carbonyl groups were introduced with the addition of modified starches, thus allowing a complete occurrence of the Maillard reaction. Resultantly, L* declined, and a* increased, leading to a more attractive color. In addition, double frying after freezing cause the shell surface to absorb more oil, resulting in higher L* higher than in unfrozen samples [59], and the changes in L* and a* were consistent with unfrozen samples. In addition, CAS and ADSP consistently enhanced a*, likely due to their acetyl and phosphate groups accelerating carbonyl-amino reactions. It was speculated that the coordination of property differences in the batter, uncontrollable changes during frying, and microstructural changes during freezing led to the color variation. Furthermore, the types and concentrations of modified starch influence the shell color to varying degrees [60]. Incorporating optimized levels of modified starches into fried, crispy meat formulations enhances color appeal and thermal stability. Compared to native starch (NS), a 10% HPDSP increased lightness (L*) by 2.3% while elevating yellowness (b*) by 13.5%, yielding a brighter golden hue. ADSP samples retained superior L* and minimized a* degradation after double frying, preserving the red-yellow balance. Freezing stability tests revealed lower L* reductions for HPDSP or ADSP, confirming their structural integrity benefits. These findings demonstrate that modified starches improve the visual appeal of fried meat by balancing brightness, minimizing over-browning, and resisting color deterioration during thermal processing. Such enhancements directly elevate consumer attractiveness and purchase intent, particularly in premium frozen snack segments that prioritize aesthetic quality and consistency.

### 3.8. T2 Relaxation Time Analysis

**Figure 6:**
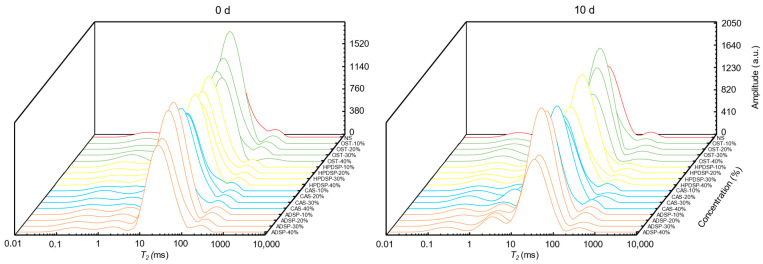
T2 inversion diagram of crisp meat with different types and concentrations of modified starches.

The changes in water state and distribution during frying and freezing are essential in predicting product stability, physiochemical changes, and shelf-life [61]. The first peak, T21 (0.1~10 ms), corresponded to water molecules bound to food through strong H-bonds. The second peak, T22 (10~100 ms), represented immobilized water strongly bonded to the monolayer water molecules. The third peak, T23 (100~1000 ms), reflected free water weakly bound to food [27]. Generally, shorter relaxation time and amplitude reveal a strong association between solids and water within a matrix, whereas long amplitude shows weak water interaction in a heterogenous system [62]. Figure 6 exhibits the water distribution in three different states of fried crisp meat. The immobilized water accounted for more than 77% of the total sample and predominated the system; thus, the relaxation time T22 and the relative area A22 were further investigated. The effect of modified starches on T22 was limited in unfrozen samples due to the limited binding time between modified starches and water. Moreover, the introduced hydrophilic groups exerted a stronger force on water molecules and bound closely to water during the freezing process [63]; hence, the T22 of the samples prepared with modified starches demonstrated a downward trend after freezing (Table 5). Despite that, the T22 of all frozen crisp meat increased compared to unfrozen samples.

The A22 of the treated groups increased compared to NS, particularly CAS-20%, demonstrating the best effect (*p* < 0.05). Furthermore, the A22 of the samples after freezing was lower than that of unfrozen crisp meat because a large amount of moisture was sublimated during frying post-freezing. Therefore, the A22 trends were consistent with T22. Nevertheless, the increasing effect of modified starch on A22 in frozen groups was stronger than in unfrozen groups, compared to NS. The findings indicated that the modified starches could maintain the water fluidity and promote the conversion of free water into immobilized water and bound water to a certain degree [64]. Starch type significantly impacts the water properties of crisp meat. CAS consistently increased immobilized water content (A22) in both unfrozen and frozen states, especially at 20% concentration. OST markedly prolonged relaxation time (T22) but showed unstable water retention after freezing. HPDSP and ADSP performed well at low-to-medium concentrations, though ADSP’s water retention declined at high concentrations post-freezing. Freezing generally increased T22 but reduced A22, with CAS partially mitigating this effect. Consequently, the water loss of pre-fried crisp meat in the frozen storage could be delayed and boost modified starches applications in commercial crisp meat production. In addition, A22 in sausage increased with the inclusion of acetate starch at 0%~5% [65]. Modified starches with hydrophilic groups enhance water binding, reducing free water and increasing bound water to optimize water–oil balance, delay moisture loss in frozen storage, and provide theoretical support for improving quality and commercial applications of pre-fried crispy meat.

### 3.9. The MRI Analysis

**Figure 7:**
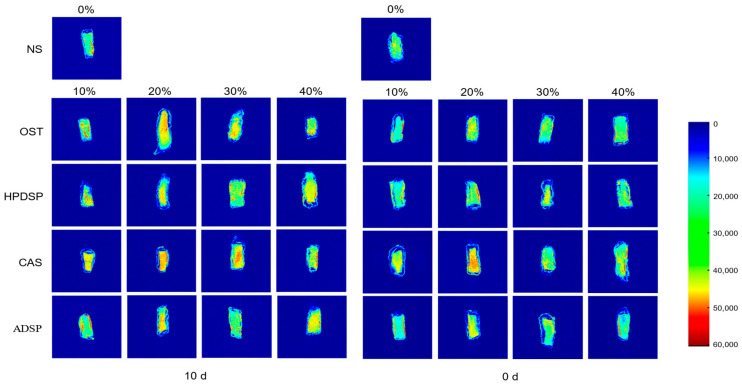
MRI images of crisp meat with different starches.

MRI is a rapid, direct, non-invasive, non-destructive, and accurate tool for food analysis, reflecting the quantity of water present and the dynamic structural characteristics of water. The proton density map allows the visualization of water distribution in samples. Generally, the proton density map and the pseudo-color picture will be brighter and redder if more hydrogen protons are within the given region, respectively [66]. The elevated A22 values (Section 3.3) correlate with restricted water mobility in cross-linked networks. As depicted in the MRI images (Figure 7), samples with 20% ADSP show intensified red regions (high proton density), confirming homogeneous water distribution that mitigates localized ice nucleation. Additionally, the degree of red and yellow decreased with the freezing process, which further demonstrated the reduction of water content. According to these findings, the combination of T2 relaxation time and MRI represented the impact of all modified starches on the moisture distribution and relative content of fried crisp meat.

## 4. Conclusions

This study quantitatively demonstrated the quality improvement effects of four modified starches on crisp meat: (1) 20% acetylated starch (ADSP) increased batter viscosity by 92% (21.5 vs. 11.2 Pa·sⁿ) and batter pickup by 18% (*p* < 0.05), with all modified starches reducing oil absorption by 12–18% during frying at 180 °C; (2) for freezing stability, ADSP-20% decreased retrogradation enthalpy (ΔHr) by 42% and increased immobilized water content (A22) to 86.3% (vs. 79.6% control) after 10-day storage at −18 °C; (3) quality preservation included maintained post-freezing crispness (24.3 N hardness vs. 18.1 N control) and controlled color difference. These results clearly identify 20% ADSP as the optimal formulation, with recommended industrial parameters: frying temperature 160–180 °C, starch replacement level 20–30%, and maximum −18 °C storage for 10 days (<15% quality loss).

Future research should focus on the following: (1) sensory evaluation to assess consumer acceptance of starch-modified crisp meat; (2) advanced freezing methods (−80 °C cryogenic vs. −18 °C) to optimize ice crystal control; (3) mixing two or more modified starches in specific proportions has become an emerging trend, but studies on the effects and mechanism of action have yet to be elucidated. These efforts will advance the precision application of modified starches in pre-fried foods.

## Figures and Tables

**Table 1:** Power low model parameters for describing flow properties of batters made from different modified starches.

Concentration (%)	OST				HPDSP				CAS				ADSP			
	K (Pa s^n^)	N (−)	*R* ^2^	*Between-Type Comparisons (p-Value)*	K (Pa s^n^)	N (−)	*R* ^2^	*Between-Type Comparisons (p-Value)*	K (Pa·s^n^)	N (−)	*R* ^2^	*Between-Type Comparisons (p-Value)*	K (Pa·s^n^)	N (−)	*R* ^2^	*Between-Type Comparisons (p-Value)*
0	10.682 ± 1.221 ^dA^	0.731 ± 0.017 ^aA^	0.9977	Reference	10.682 ± 1.221 ^bcA^	0.731 ± 0.017 ^bcA^	0.9977	1.000 (K), 1.000 (n)	10.682 ± 1.221 ^bA^	0.731 ± 0.017 ^bA^	0.9977	1.000 (K), 1.000 (n)	10.682 ± 1.221 ^cA^	0.731 ± 0.017 ^bA^	0.9977	1.000 (K), 1.000 (n)
10	15.982 ± 0.934 ^bA^	0.671 ± 0.017 ^bB^	0.9961		8.361 ± 1.171 ^cC^	0.781 ± 0.038 ^aA^	0.9941	<0.001 (K), <0.001 (n)	13.915 ± 0.750 ^aB^	0.694 ± 0.012 ^cB^	0.9895	0.012 (K), 0.342 (n)	15.660 ± 2.037 ^bA^	0.686 ± 0.0185 ^cB^	0.986	0.843 (K), 0.521 (n)
20	19.077 ± 1.505 ^aAB^	0.669 ± 0.020 ^bB^	0.9969		13.797 ± 1.237 ^abBC^	0.701 ± 0.019 ^cAB^	0.9942	<0.001 (K), 0.187 (n)	11.866 ± 0.989 ^bC^	0.710 ± 0.021 ^bcA^	0.9989	<0.001 (K), 0.072 (n)	21.473 ± 3.716 ^aA^	0.638 ± 0.033 ^dC^	0.9876	0.432 (K), 0.421 (n)
30	13.382 ± 1.255 ^cC^	0.726 ± 0.019 ^aA^	0.9982		14.264 ± 0.495 ^aB^	0.712 ± 0.008 ^cB^	0.9952	0.342 (K), 0.521 (n)	14.052 ± 1.294 ^aB^	0.688 ± 0.022 ^cC^	0.9965	0.621 (K), 0.112 (n)	15.716 ± 0.705 ^bA^	0.677 ± 0.009 ^cC^	0.9949	0.087 (K), 0.034 (n)
40	11.107 ± 0.679 ^dA^	0.733 ± 0.020 ^aA^	0.9996		9.964 ± 1.822 ^cB^	0.758 ± 0.032 ^abA^	0.9977	0.421 (K), 0.521 (n)	8.800 ± 0.104 ^cC^	0.765 ± 0.008 ^aA^	0.9989	0.012 (K), 0.342 (n)	7.893 ± 0.421 ^cC^	0.776 ± 0.021 ^aA^	0.9988	<0.001 (K), 0.087 (n)

0% indicates native starch. K and n indicate consistency coefficient and flow behavior index (dimensionless), respectively; R2 indicates determination coefficient. Lowercase letters (^a–d^): Significant differences within columns (same starch type, different concentrations, *p* < 0.05). Uppercase letters (^A–C^): Significant differences within rows (same concentration, different starch types, *p* < 0.05).

**Table 2:** Moisture content (%) of fried and twice-fried crisp meat.

Concentration (%)	OST		HPDSP		CAS		ADSP	
	0 d	10 d	0 d	10 d	0 d	10 d	0 d	10 d
0	58.92 ± 0.55 ^bA^	56.72 ± 2.71 ^bA^	58.92 ± 0.55 ^bA^	56.72 ± 2.71 ^cdA^	58.92 ± 0.55 ^cdA^	56.72 ± 2.71 ^cA^	58.92 ± 0.55 ^cA^	56.72 ± 2.71 ^abA^
10	58.87 ± 0.45 ^bC^	56.22 ± 0.38 ^bC^	58.27 ± 0.49 ^bC^	57.90 ± 0.94 ^bcB^	60.82 ± 0.96 ^bA^	60.33 ± 0.74 ^aA^	59.98 ± 0.72 ^bB^	56.17 ± 0.94 ^abC^
20	60.46 ± 0.70 ^aB^	59.36 ± 0.25 ^aA^	60.62 ± 0.81 ^aB^	59.66 ± 0.31 ^aA^	62.58 ± 0.92 ^aA^	58.42 ± 0.70 ^bB^	61.08 ± 0.17 ^aB^	58.29 ± 0.43 ^aB^
30	58.31 ± 0.72 ^bAB^	53.14 ± 0.34 ^cC^	59.07 ± 0.66 ^bA^	58.54 ± 0.45 ^bA^	58.22 ± 0.97 ^dB^	56.30 ± 0.82 ^cB^	58.80 ± 0.52 ^cAB^	55.58 ± 0.82 ^bB^
40	55.08 ± 0.74 ^cD^	53.72 ± 0.45 ^cC^	58.99 ± 0.51 ^bC^	56.26 ± 0.35 ^dA^	59.68 ± 0.34 ^cB^	55.03 ± 0.83 ^dB^	61.62 ± 0.43 ^aA^	54.92 ± 0.42 ^bB^

0% indicates native starch. Lowercase letters (^a–d^): Significant differences within columns (same starch type, same freezing time, different concentrations, *p* < 0.05). Uppercase letters (^A–D^): Significant differences within rows (same concentration, same freezing time, different starch types, *p* < 0.05).

**Table 3:** Oil content (%) of fried and twice-fried crisp meat.

Concentration (%)	OST		HPDSP		CAS		ADSP	
	0 d	10 d	0 d	10 d	0 d	10 d	0 d	10 d
0	17.90 ± 0.26 ^aA^	19.94 ± 1.21 ^cA^	17.90 ± 0.26 ^aA^	19.94 ± 1.21 ^bA^	17.90 ± 0.26 ^abA^	19.94 ± 1.21 ^cA^	17.90 ± 0.26 ^abA^	19.94 ± 1.21 ^aA^
10	15.50 ± 1.31 ^bB^	20.51 ± 0.11 ^bcA^	16.54 ± 2.54 ^abAB^	18.82 ± 1.34 ^cB^	17.85 ± 1.29 ^abA^	18.74 ± 1.43 ^cB^	17.05 ± 1.44 ^abAB^	21.04 ± 1.48 ^aA^
20	11.86 ± 0.71 ^cC^	17.88 ± 1.58 ^dB^	14.51 ± 0.23 ^bB^	18.55 ± 0.38 ^cB^	16.44 ± 1.70 ^bA^	19.86 ± 2.22 ^cA^	16.57 ± 1.53 ^bA^	19.49 ± 0.16 ^aA^
30	18.68 ± 1.34 ^aA^	23.28 ± 1.34 ^aA^	16.61 ± 1.29 ^abB^	19.51 ± 0.75 ^bcC^	19.49 ± 0.69 ^aA^	22.97 ± 0.31 ^aA^	19.51 ± 2.08 ^aA^	21.33 ± 0.40 ^aB^
40	19.01 ± 1.70 ^aA^	21.42 ± 0.23 ^bA^	16.96 ± 2.06 ^abB^	21.30 ± 0.13 ^aA^	17.48 ± 1.14 ^bAB^	21.56 ± 0.24 ^bA^	17.20 ± 0.85 ^abAB^	20.91 ± 2.50 ^aA^

0% indicates native starch. Lowercase letters (^a–d^): Significant differences within columns (same starch type, same freezing time, different concentrations, *p* < 0.05). Uppercase letters (^A–C^): Significant differences within rows (same concentration, same freezing time, different starch types, *p* < 0.05).

**Table 4:** Color parameters of crisp meat. (**A**): L*, a*, and b* values of unfrozen samples; (**B**): L*, a*, and b* values of samples frozen for 10 d.

**(A)**												
**Concentration (%)**	**OST**			**HPDSP**			**CAS**			**ADSP**		
	L*	a*	b*	L*	a*	b*	L*	a*	b*	L*	a*	b*
0	51.40 ± 1.08 ^aA^	7.82 ± 0.51 ^cA^	26.74 ± 1.22 ^abA^	51.40 ± 1.08 ^aA^	7.82 ± 0.51 ^bA^	26.74 ± 1.22 ^bcA^	51.40 ± 1.08 ^aA^	7.82 ± 0.51 ^cA^	26.74 ± 1.22 ^cA^	51.40 ± 1.08 ^aA^	7.82 ± 0.51 ^cA^	26.74 ± 1.22 ^dA^
10	47.03 ± 0.81 ^bcBC^	9.71 ± 0.85 ^bB^	25.89 ± 0.42 ^bC^	50.45 ± 2.54 ^abA^	9.69 ± 0.64 ^aB^	29.14 ± 1.55 ^aAB^	48.98 ± 1.30 ^bAB^	11.21 ± 0.99 ^aA^	30.30 ± 1.25 ^abA^	46.73 ± 1.03 ^cC^	10.77 ± 0.78 ^bAB^	27.70 ± 0.96 ^cBC^
20	48.39 ± 0.62 ^bB^	10.86 ± 1.06 ^aAB^	27.53 ± 1.47 ^aB^	50.53 ± 1.75 ^abA^	9.57 ± 1.07 ^aB^	26.78 ± 0.70 ^bcB^	50.59 ± 1.15 ^abA^	10.43 ± 0.86 ^bAB^	30.02 ± 1.90 ^bA^	48.81 ± 2.16 ^bB^	11.60 ± 1.37 ^aA^	30.14 ± 0.97 ^aA^
30	46.12 ± 0.75 ^cB^	10.53 ± 0.75 ^aA^	27.44 ± 1.06 ^aC^	50.14 ± 0.89 ^abA^	9.98 ± 0.85 ^aA^	27.06 ± 1.08 ^bC^	49.94 ± 1.43 ^bAB^	10.46 ± 0.86 ^bA^	31.21 ± 1.32 ^aA^	48.61 ± 2.33 ^bAB^	10.5 ± 0.73 ^bA^	28.94 ± 0.89 ^bB^
40	47.15 ± 0.96 ^bcB^	9.53 ± 0.65 ^bB^	26.47 ± 1.11 ^abC^	49.08 ± 0.83 ^bAB^	7.95 ± 1.12 ^bC^	26.19 ± 0.89 ^cC^	49.83 ± 1.25 ^bA^	10.39 ± 0.50 ^bA^	30.25 ± 1.08 ^abA^	48.58 ± 1.23 ^bAB^	7.95 ± 0.75 ^cC^	27.72 ± 1.11 ^cB^
**(B)**												
**Concentration (%)**	**OST**			**HPDSP**			**CAS**			**ADSP**		
	***L*** *	***a*** *	***b*** *	***L*** *	***a*** *	***b*** *	***L*** *	***a*** *	***b*** *	***L*** *	***a*** *	***b*** *
0	53.92 ± 0.37 ^aA^	7.34 ± 0.78 ^cA^	26.86 ± 0.66 ^bA^	53.92 ± 0.37 ^aA^	7.34 ± 0.78 ^bA^	26.86 ± 0.66 ^bA^	53.92 ± 0.37 ^aA^	7.34 ± 0.78 ^dA^	26.86 ± 0.66 ^cA^	53.92 ± 0.37 ^aA^	7.34 ± 0.78 ^dA^	26.86 ± 0.66 ^cA^
10	51.40 ± 0.93 ^bA^	9.27 ± 0.75 ^bA^	27.34 ± 0.38 ^abAB^	50.92 ± 1.99 ^bA^	7.43 ± 0.34 ^bB^	25.47 ± 0.43 ^cC^	49.93 ± 1.22 ^bcAB^	8.40 ± 1.22 ^cAB^	26.38 ± 1.22 ^cBC^	47.72 ± 1.45 ^cB^	9.57 ± 0.91 ^bA^	28.37 ± 0.89 ^bA^
20	50.49 ± 0.80 ^bcA^	10.68 ± 0.48 ^aAB^	28.06 ± 0.86 ^aC^	50.45 ± 1.92 ^bA^	8.47 ± 1.05 ^aC^	28.42 ± 0.99 ^aBC^	50.78 ± 1.12 ^bA^	9.32 ± 0.97 ^bBC^	29.43 ± 1.25 ^aAB^	50.51 ± 0.88 ^bA^	11.11 ± 1.27 ^aA^	30.36 ± 1.42 ^aA^
30	47.13 ± 0.76 ^dB^	10.45 ± 0.60 ^aA^	24.75 ± 1.32 ^cB^	50.28 ± 1.19 ^bcA^	7.58 ± 1.22 ^bC^	23.67 ± 1.48 ^dB^	50.13 ± 2.01 ^bcA^	9.98 ± 0.89 ^aAB^	28.35 ± 0.85 ^bA^	51.04 ± 1.89 ^bA^	9.44 ± 0.81 ^bB^	27.36 ± 0.86 ^bcA^
40	49.33 ± 1.44 ^cdAB^	9.28 ± 0.78 ^bA^	27.36 ± 0.62 ^abA^	50.29 ± 1.18 ^bAB^	5.32 ± 0.64 ^cC^	21.48 ± 1.29 ^eB^	49.73 ± 1.64 ^cB^	9.90 ± 0.69 ^aA^	28.52 ± 0.81 ^bA^	51.32 ± 1.97 ^bA^	8.30 ± 0.80 ^cB^	28.25 ± 1.07 ^bA^

L*, a*, and b* reflect lightness from black (0) to white (100), green (−) to red (+), and blue (−) to yellow (+), respectively. 0% indicates native starch. Lowercase letters (^a–e^): Significant differences within columns (same starch type, different concentrations, *p* < 0.05). Uppercase letters (^A–C^): Significant differences within rows (same concentration, different starch types, *p* < 0.05).

**Table 5:** Relaxation time T22 and relative area A22 of crisp meat. (**A**): T22 and A22 of unfrozen samples; (**B**): T22 and A22 of samples frozen for 10 d.

(**A**)								
**Concentration (%)**	**OST**		**HPDSP**		**CAS**		**ADSP**	
	***T*** **_22_** **(ms)**	***A*** **_22_** **(%)**	***T*** **_22_** **(ms)**	***A*** **_22_** **(%)**	***T*** **_22_** **(ms)**	***A*** **_22_** **(%)**	***T*** **_22_** **(ms)**	***A*** **_22_** **(%)**
0	21.54 ± 04 ^cA^	85.74 ± 1.13 ^cA^	21.54 ± 06 ^bcA^	85.74 ± 1.13 ^cA^	21.54 ± 03 ^abA^	85.74 ± 1.13 ^dA^	21.54 ± 01 ^dA^	85.74 ± 1.13 ^cA^
10	26.01 ± 2.14 ^aA^	88.38 ± 0.88 ^aA^	22.62 ± 1.86 ^abB^	86.65 ± 0.89 ^bB^	18.74 ± 1.61 ^cC^	88.09 ± 0.88 ^bcA^	26.01 ± 2.14 ^aC^	87.93 ± 1.28 ^abA^
20	23.16 ± 2.28 ^bAB^	88.57 ± 2.01 ^aB^	20.61 ± 1.62 ^cB^	87.63 ± 0.66 ^aB^	22.35 ± 02 ^aAB^	90.70 ± 0.41 ^aA^	24.77 ± 04 ^bA^	88.49 ± 0.80 ^aB^
30	21.54 ± 05 ^cA^	87.67 ± 0.64 ^abA^	23.16 ± 1.86 ^aA^	87.07 ± 1.18 ^abA^	22.62 ± 1.86 ^aA^	88.84 ± 1.43 ^bA^	23.16 ± 1.86 ^cA^	87.37 ± 1.31 ^bA^
40	20.14 ± 1.98 ^dB^	86.39 ± 0.45 ^bA^	23.16 ± 1.86 ^aA^	87.48 ± 0.99 ^aA^	19.67 ± 1.62 ^bB^	86.92 ± 1.09 ^cA^	23.70 ± 1.86 ^bcA^	87.97 ± 0.99 ^bA^
(**B**)								
**Concentration (%)**	**OST**		**HPDSP**		**CAS**		**ADSP**	
	***T*** **_22_** **(ms)**	***A*** **_22_** **(%)**	***T*** **_22_** **(ms)**	***A*** **_22_** **(%)**	***T*** **_22_** **(ms)**	***A*** **_22_** **(%)**	***T*** **_22_** **(ms)**	***A*** **_22_** **(%)**
0	33.97 ± 2.45 ^aA^	79.55 ± 0.94 ^cA^	33.97 ± 2.45 ^aA^	79.55 ± 0.94 ^dA^	33.97 ± 2.45 ^aA^	79.55 ± 0.94 ^dA^	33.97 ± 2.45 ^aA^	79.55 ± 0.94 ^cA^
10	26.35 ± 5.77 ^cAB^	80 ± 4.45 ^bcB^	31.32 ± 2.46 ^abA^	84.87 ± 1.13 ^bA^	23.96 ± 1.61 ^cB^	85.28 ± 0.37 ^bA^	27.24 ± 2.14 ^cAB^	84.87 ± 1.13 ^bA^
20	29.90 ± 2.46 ^bA^	86.25 ± 1.80 ^aB^	30.61 ± 2.46 ^bA^	87.71 ± 2.20 ^aB^	28.62 ± 3.26 ^bcA^	91.47 ± 0.60 ^aA^	25.70 ± 1.85 ^dB^	86.17 ± 2.62 ^aB^
30	34.38 ± 2.83 ^aA^	82.27 ± 0.98 ^bA^	28.48 ± 01 ^cB^	82.99 ± 0.97 A^c^	29.55 ± 2.13 ^bB^	82.22 ± 0.90 ^cA^	30.61 ± 2.46 ^bB^	78.98 ± 3.09 ^cB^
40	26.01 ± 2.14 ^cBC^	77.72 ± 2.69 ^cB^	28.62 ± 3.26 ^bcAB^	84.20 ± 1.02 ^bA^	23.70 ± 1.86 ^cC^	85.57 ± 0.91 ^bA^	30.61 ± 2.46 ^bA^	78.70 ± 0.94 ^cB^

T22 indicates the relaxation time of immobilized water; A22 indicates the relative area of immobilized water. 0% indicates native starch. Lowercase letters (^a–d^): Significant differences within columns (same starch type, different concentrations, *p* < 0.05). Uppercase letters (^A–C^): Significant differences within rows (same concentration, different starch types, *p* < 0.05).

## Data Availability

The original contributions presented in this study are included in the article. Further inquiries can be directed to the corresponding author.

## Abbreviations

The following abbreviations are used in this manuscript:

OST	Oxidized starch
HPDSP	hydroxypropyl distarch phosphate
CAS	starch acetate
ADSP	acetylated distarch phosphate
NS	native starch
